# Hyperglycemia may increase deep vein thrombosis in trauma patients with lower limb fracture

**DOI:** 10.3389/fcvm.2022.944506

**Published:** 2022-09-08

**Authors:** Xiaojie Liu, Tiajun Li, Hui Xu, Chunhua Wang, Xiaojun Ma, Hui Huang, Yanling Hu, Haichen Chu

**Affiliations:** ^1^Department of Anestheiology, The Affiliated Hospital of Qingdao University, Qingdao, China; ^2^Department of Oncology, The Affiliated Hospital of Qingdao University, Qingdao, China; ^3^Department of Orthopedic, The Affiliated Hospital of Qingdao University, Qingdao, China

**Keywords:** hyperglycemia, deep vein thrombosis, trauma, glucose, immobilization

## Abstract

Diabetes mellitus is associated with prothrombotic states and thrombotic events. This study examined the association between preoperative glucose levels and deep vein thrombosis (DVT) in trauma patients undergoing surgery for lower limb fracture. Data from 1,591 patients who underwent fracture surgery between January 2017 and March 2022 at the Affiliated Hospital of Qingdao University were queried from institutional electronic medical records. A total study population of 1,086 patients was identified, comprising 138 patients who experienced DVT and 948 controls. The primary outcome was DVT. Multiple logistic regression analyses were performed and a receiver operating characteristic (ROC) curve was generated. Age, D-dimer level, preoperative RBC count, and preoperative glucose level were independent predictors of DVT. The two highest categories of D-dimer level (≥ 960, < 2,102; ≥ 2,102 ng/ml) increased the odds ratio for DVT by 4.215 times [95% confidence interval (CI) 1.820–9.761] and 7.896 times (95% CI 3.449–18.074), respectively, compared with the lowest reference category (< 490 ng/ml). The area under the curve (AUC) for the preoperative glucose level was 0.605. Hyperglycemia (glucose ≥ 6.1, < 7.0 mmol/l) increased the odds of DVT by 1.889-fold [95% CI (1.085–3.291); *p* < 0.0001] compared with euglycemia (glucose < 6.1 mmol/l). We therefore observed an association between preoperative hyperglycemia and DVT in patients with lower limb fractures. There are several modalities for controlling hyperglycemia, offering potential targets for future improvement.

## Introduction

As the focus on enhanced recovery after surgery increases, more attention is being given to patients with orthopedic diseases, especially preoperative deep vein thrombosis (DVT) (1).

Once a fracture has occurred, immobilization is necessary. However, immobilization reduces blood flow and damages endothelial cells. In a context of abnormal coagulation in the deep veins, this could lead to the formation of DVT, potentially resulting in fatal pulmonary embolism (PE).

The prevalence of perioperative DVT is as high as 50–60% in fracture patients. In addition to trauma or fracture, age, sex, smoking status, congestive heart failure, cancer, obesity, oral contraceptive use, recent surgery, and history of previous venous thromboembolism (VTE) are commonly thought to be risk factors (2).

Previous studies have found age, male sex, coronary artery disease, more than 6 days prior to surgery, chronic renal insufficiency, smoking status, time from injury to ultrasonography examination, platelet count (PLT), and D-dimer at admission to be risk factors for perioperative DVT in patients with fracture (3–5).

Another study observed an overall incidence of postoperative DVT of 18.91% in patients with thoracolumbar fractures. Fibrinogen level (FIB) and D-dimer are generally considered risk factors for DVT (2). A large administrative database study suggested that overweight and obesity increased risk of developing PE, but not DVT, after primary total hip or knee arthroplasty (6).

Our previous study illustrated that obesity increases insulin resistance, which can increase chronic inflammation. A chronic low-grade inflammatory state is associated with increased plasma levels of inflammatory markers (7). Another previous study found that preoperative C-reactive protein (CRP), as a marker of inflammation, was also associated with body mass index (BMI), activated partial thromboplastin time, and fibrinogen levels (8). Other studies have shown that patients with COVID-19 admitted with hyperinsulinemia, hyperglycemia, and hypertension had increased inflammation, coagulation, and thrombosis risk (9), and that hyperglycemia potentiated the prothrombotic effect of aldosterone in a rat model of arterial thrombosis (10). However, no studies have shown an association between glucose level and thrombosis, especially DVT, in fracture patients.

Therefore, this study aims to illustrate the association between preoperative glucose level and DVT in patients experiencing lower extremity fracture and its specificity and sensitivity for diagnosis.

## Subjects and methods

### Study population

This was a single-center retrospective study of consecutive patients suffering from limb fracture who accepted surgery at the Affiliated Hospital of Qingdao University. The study was approved by the Ethics Committee of the Affiliated Hospital of Qingdao University. The study was conducted in accordance with the Helsinki Declaration and following the Strengthening the Reporting of Cohort Studies in Surgery (STROCSS) guidelines.

Data were collected from hospital electronic medical records. Patients 18 years of age or older who were admitted to the Affiliated Hospital of Qingdao University in Qingdao, China, between January 2017 and March 2022 for limb fracture, and who underwent preoperative examination for DVT of the bilateral lower extremities, were deemed eligible. We excluded patients with older fractures (> 21 days from injury); patients with a history of cancer, VTE, or peripheral vascular disease; patients who had undergone recent anticoagulant and antiplatelet therapy (such as aspirin, ticagrelor, warfarin, heparin, low-molecular-weight heparin, or others); patients who had used lower extremity compressive devices after injury; patients with no documentation of any DVT examination; and patients with incomplete medical records. Lower limb fractures were defined as hip, femur, tibia/fibula, ankle, or foot fractures. A total of 1,591 patients were identified.

### Data collection

Patients’ basic characteristics, history of chronic diseases, preoperative laboratory white blood cell (WBC) count, coagulation parameters [platelet (PLT) count, red blood cell (RBC) count, prothrombin time (PT), activated partial thromboplastin time (APTT), D-dimer, fibrinogen (FIB), thrombin time (TT), platelet distribution width (PDW)], mean corpuscular volume (MCV), and mean platelet volume (MPV) were recorded.

A duplex ultrasonography (DUS) scan of bilateral lower extremity veins (common femoral vein, superficial femoral vein, deep femoral vein, popliteal vein, anterior tibial vein, posterior tibial vein, and peroneal vein) was performed to determine the presence of DVT.

Deep vein thrombosis was diagnosed according to the Guidelines for the Diagnosis and Treatment of Deep Vein Thrombosis (3rd Edition) proposed by the Chinese Medical Association (11). The diagnostic criteria were loss of or no compressibility of the vein, lumen obstruction or filling defect, lack of respiratory variation in above-knee vein segments, and inadequate flow augmentation to calf and foot with compression maneuvers.

Hyperglycemia and euglycemia (normal glucose) were defined as glucose ≥ 6.1 mmol/L and glucose < 6.1 mmol/L, respectively. We further categorized the patients into three groups according to fasting blood glucose level (12).

### Statistical analysis

Categorical variables are expressed as numbers and percentages, and continuous variables are expressed as the mean ± standard deviation (for normally distributed continuous variable comparisons between groups) and the median (for non-normally distributed continuous variable comparisons between groups). To compare the characteristics between cohorts, we used the χ^2^ test for categorical variables and Student’s *t*-test or the Wilcoxon rank-sum test for continuous variables based on the distributions.

Simple logistic regression was used to analyze the relationship between demographic data and DVT. A multiple logistic regression model was used to identify the independent variables associated with DVT. For both models, covariates were selected into the logistic regression models if the *p*-value was less than 0.1 after univariate regression analysis. The enter method was applied to build the multiple logistic regression model. The goodness of fit is illustrated by a Nagelkerke r-square of 0.212 and c-statistic of 0.786 (95% CI 0.749, 0.824).

Based on preoperative glucose levels, we divided patients into two groups by comparing baseline levels. An index of *p* < 0.1 was incorporated into the multivariate regression analysis. Statistical significance was defined as *p* < 0.05.

All statistical analyses were performed with SPSS version 23.0 software (SPSS Inc., Chicago, IL, United States).

## Results

**FIGURE 1:**
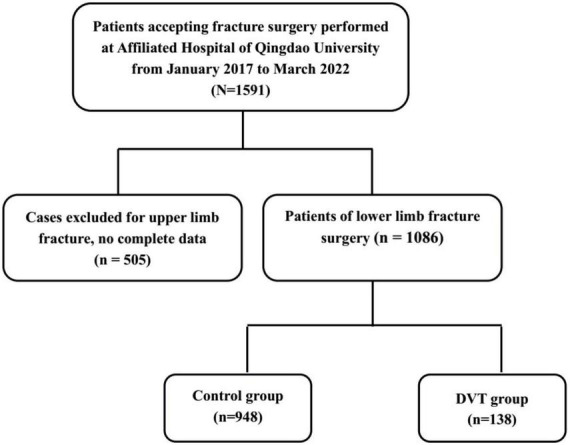
A flowchart depicting the selection of the study population. A group of 1,591 patients undergoing fracture surgery was screened. After excluding 505 patients, a total of 1,086 patients with lower limb fractures and complete information were included, comprising 138 patients suffering from DVT and 948 controls.

Initially, 1,591 patients meeting the eligibility criteria were identified. A total of 505 patients were then excluded due to incomplete information and presence of upper limb fracture. After screening, the total study population comprised 1,086 patients, including 138 patients suffering from DVT and 948 controls (Figure 1).

Patients’ demographic and clinical data are presented in Table 1. Statistically significant differences between DVT and control groups in terms of age, alcohol consumption, hypertension, diabetes, cerebral disease, preoperative glucose levels, neutrophil levels, red blood cell counts, hemoglobin levels, FIB, D-dimer medians, and PT were noted (*p* < 0.05).

**TABLE 1:** Univariate analysis of factors associated with preoperative DVT (*n* = 1,086).

Characteristic	Control (*n* = 948)	DVT (*n* = 138)	*P*-value
Age	55.4 ± 16.8	65.8 ± 14.1	0.002
Male sex, *n* (%)	494 (55.32)	68 (50.37)	0.534
BMI, medians (IQRs)	24.4 (22.1–27.2)	23.9 (22–26.6)	0.300
Smoking, *n* (%)	200 (22.40)	25 (18.52)	0.403
Alcohol consumption, *n* (%)	184 (20.60)	25 (18.52)	< 0.001
Hypertension, *n* (%)	207 (23.18)	47 (34.81)	0.002
Chronic lung disease, *n* (%)	29 (3.25)	5 (3.70)	0.722
Arrhythmia, *n* (%)	15 (1.68)	1 (0.74)	0.377
CHD, *n* (%)	73 (8.17)	10 (7.41)	0.851
Peripheral vascular disease, *n* (%)	1 (0.11)	1 (0.74)	0.239
Diabetes, *n* (%)	101 (11.31)	27 (20.00)	0.003
Cerebral disease, *n* (%)	36 (4.03)	12 (8.89)	0.009
Surgery history, *n* (%)	333 (37.29)	50 (37.04)	0.616
Cr	84.2 ± 20.8	85.9 ± 30.7	0.531
Glucose, median (IQRs), mmol/L	5.63 (5.06–6.60)	6.05 (5.36–7.23)	< 0.001
WBC (10^9^/L)	7.98 ± 2.57	8.32 ± 2.51	0.179
Neutrophils (10^9^/L)	5.61 ± 2.90	6.13 ± 2.72	0.038
PLT	212.6 ± 69.5	214.2 ± 74.4	0.819
RBC	4.16 ± 0.65	3.70 ± 0.66	< 0.001
HGB	126.1 ± 21.2	113.2 ± 19.7	< 0.001
MCHC	337.0 ± 12.3	337.3 ± 12.3	0.795
MCH	30.4 ± 2.2	30.7 ± 2.1	0.091
MCV	90.2 ± 5.3	91.1 ± 4.9	0.046
MPV	10.38 ± 0.87	10.39 ± 0.88	0.934
PDW	11.94 ± 1.89	11.77 ± 1.82	0.325
FIB	3.39 ± 1.01	3.80 ± 1.15	< 0.001
D-dimer median (IQRs), ng/ml	850 (470–1910)	2,120 (1,180–4,155)	< 0.001
PT (s)	11.86 ± 1.28	12.23 ± 1.65	0.013
APTT (s)	30.49 ± 6.26	30.89 ± 6.24	0.487
TT (s)	16.69 ± 1.39	16.34 ± 1.31	0.005

CHD, coronary heart disease; BMI, body mass index; HGB, hemoglobin; PLT, platelet; PT, prothrombin time; APTT, activated partial thromboplastin time; TT, thrombin time; FIB, fibrinogen; PDW, platelet distribution width; MCV, mean corpuscular volume; WBC, white blood cells; RBC, red blood cells; MPV, mean platelet volume; IQRs, interquartile ranges.

Younger age; the absence of hypertension, diabetes, or cerebral events; higher levels of, hemoglobin, and preoperative RBC; lower levels of neutrophils, FIB, TT, and PT; and lower D-dimer medians and preoperative glucose were significantly negatively correlated with preoperative DVT (*p* < 0.05).

Table 2 presents the results of the univariate and multiple logistic regression. The model is adjusted for age, hypertension, diabetes, cerebral events, glucose, neutrophils, HGB, RBC, PT, TT, FIB, and D-dimers. Variables with no statistical significance have been omitted from the table. Older age, lower preoperative RBC, and higher D-dimer medians and preoperative glucose levels were independent predictors of DVT (*p* < 0.05). However, for preoperative RBC, the odds ratio is only 0.639 (95% CI 0.469–0.870). Compared to the lowest reference category of D-dimer (< 490 ng/ml), the two highest categories of D-dimer levels (≥ 960, < 2,102; ≥ 2,102 ng/ml) increased the odds ratio for DVT by 4.215 times (95% CI 1.820–9.761) and 7.896 times (95% CI 3.449–18.074), respectively.

**TABLE 2:** Risk factors associated with DVT by univariate and multivariate analyses.

DVT	Univariate analyses		Multivariate analyses	
	Crude OR (95% CI)	*P*-value	Adjusted OR* (95% CI)	*P*-value
Age	1.041 (1.029–1.053)	< 0.001	1.021 (1.008–1.035)	0.002
Male sex	0.893 (0.625–1.276)	0.534		
Smoking	0.824 (0.520–1.306)	0.410		
Hypertension	1.849 (1.259–2.715)	0.002		
Diabetes	2.033 (1.272–3.247)	0.003		
Cerebral events	2.410 (1.222–4.754)	0.011		
BMI	0.980 (0.938–1.024)	0.366		
Glucose	1.142 (1.066–1.223)	< 0.001	1.098 (1.013–1.191)	0.024
Neutrophils	1.076 (1.009–1.148)	0.027		
HGB	0.975 (0.968–0.983)	< 0.001		
RBC	0.370 (0.284–0.482)	< 0.001	0.639 (0.469–0.870)	0.004
PLT	1.000 (0.998–1.003)	0.803		
PT	1.189 (1.056–1.337)	0.004		
APTT	1.010 (0.982–1.038)	0.487		
TT	0.845 (0.744–0.980)	0.010		
FIB	1.409 (1.203–1.651)	< 0.001		
**D-dimers (ng/ml)**				
1 (< 490)	Ref		Ref	
2 (≥ 490, < 960)	2.841 (1.148–7.030)	0.024	2.250 (0.896–5.652)	0.084
3 (≥ 960, < 2102)	6.503 (2.873–14.719)	< 0.001	4.215 (1.820–9.761)	0.001
4 (≥ 2102)	13.731 (6.188–30.468)	< 0.001	7.896 (3.449–18.074)	< 0.001

BMI, body mass index; HGB, hemoglobin; PLT, platelet; PCT, plateletcrit; PT, prothrombin time; APTT, activated partial thromboplastin time; TT, thrombin time; FIB, fibrinogen; PDW, platelet distribution width; MCV, mean corpuscular volume; WBC, white blood cells; RBC, red blood cells; MPV, mean platelet volume.

*Adjusted by age, hypertension, diabetes, cerebral events, glucose, neutrophils, HGB, RBC, PT, TT, FIB, and D-dimer.

**FIGURE 2:**
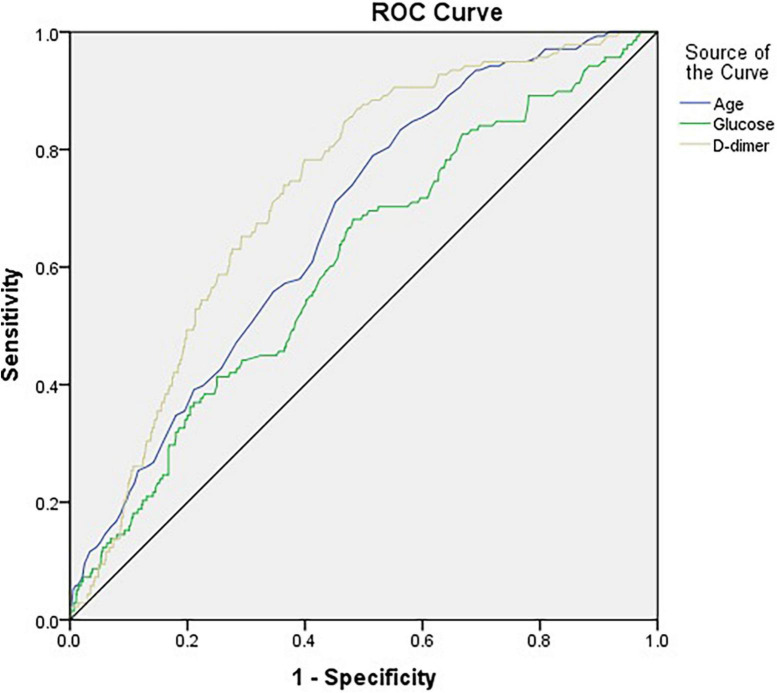
The horizontal axis indicates the 1-specificity, and the vertical axis indicates the sensitivity of each variable in predicting DVT. The area under the curve (AUC) represents the respective ability to discriminate DVT cases.

Figure 2 and Table 3 illustrate the AUC and ROC (receiver operating characteristic curve) analysis. Age, preoperative glucose level, and D-dimer levels were found to be statistically significant (AUC, 0.672; 95% CI, 0.629–0.716; *p* < 0.001; AUC, 0.605; 95% CI, 0.555–0.655; *p* < 0.001; AUC, 0.728; 95% CI, 0.688–0.768; *p* < 0.001, respectively).

**TABLE 3:** The ROC and AUC to determine the optimal cut-off value for each variable.

Variable	AUC	95% CI		*P*-value
		Lower limit	Upper limit	
Age	0.672	0.629	0.716	< 0.001
Glucose	0.605	0.555	0.655	< 0.001
D-dimer	0.728	0.688	0.768	< 0.001

Subsequently, patients’ fasting glucose concentrations were categorized into quantiles.

This revealed a weakly increased risk, if at all, with a 1.889-fold (95% CI 1.085–3.291) increased risk of DVT in the middle category compared with the lowest reference category (Table 4). Self-reported diabetes was not associated with an increased risk of DVT (Table 2).

**TABLE 4:** Fasting glucose levels categorized by diabetes mellitus diagnosis according to World Health Organization criteria (WHO/IDF 2019) and the risk of a first DVT event.

Glucose level (mmol/L)	Patients	Odds ratio (95% CI)	Adjusted odds ratio* (95% CI)
< 6.1	666	Ref	Ref
=6.1, < 7.0	215	1.900 (1.198–3.259)	1.889 (1.085–3.291)
=7.0	205	0.921 (0.568–1.494)	1.092 (0.665–1.794)

*Adjusted by fibrinogen and D-dimer.

## Discussion

This study illustrated that a high preoperative glucose level was independently correlated with preoperative DVT, in addition to the usual risk factors.

### Immobilization as a risk factor

The patients included in this study suffered from fracture and therefore required immobilization. When a limb is immobilized, blood flow slows down, which can cause a hypercoagulable state in the body. This is consistent with the study of Wells’ 10-item deep vein thrombosis score, which is the most frequently used score in clinical practice for patients with suspected DVT. The scoring criteria include items such as active cancer, paralysis, paresis, recent plaster cast on the lower extremities, recent immobilization > 3 days or major surgery within the past 4 weeks, and localized tenderness of the deep venous system (13). However, because our data were extracted from electronic medical records, the time from injury to ultrasonography examination was not accurately calculated. The low number of patients in the DVT group is low as it occurred in Asia, not Europe and America; ethnic characteristics may have contributed to these differences.

### Age as a risk factor

A previous study illustrated that age is an important risk factor for developing thrombotic cardiovascular complications, both in the arterial (acute myocardial infarction, stroke) and venous (deep vein thrombosis, pulmonary embolism) systems (14). A previous study by our research group also illustrated that older age increases perioperative thrombosis events after coronary artery bypass grafting (15). Aging in humans is associated with heightened plasma levels of coagulation proteins (e.g., factor VII, factor VIII, and fibrinogen) or antifibrinolytic factors [e.g., plasminogen activator inhibitor (PAI)-1].

Almost all fracture studies have identified age as a risk factor, which was also confirmed by this study. This study also illustrated that age is a risk factor for perioperative DVT in patients with fracture (odds ratio 1.019, 95% CI 1.005–1.033; *p* = 0.006) (16).

### Fibrinogen as a risk factor

Fibrinogen has become known as a systemic biomarker of inflammation, similar to CRP, tumour necrosis factor-α, and interleukin (17). Levels of fibrinogen were correlated with CRP after adjustment for potential confounders, which has also been demonstrated in our previous studies (8). Furthermore, elevated circulating fibrinogen levels are associated with an increased risk of cardiovascular disease, such as coronary heart disease and stroke (18, 19). Fibrinogen levels also predict the incidence of DVT (20, 21). A recent study illustrated the novel molecular mechanisms: selective reduction of fibrinogen may limit thrombosis while preserving haemostatic functions (22). Vein thrombosis in pregnancy patients increased significantly compared to the control group (23). However, this report did not discuss a correlation between fibrinogen and DVT. More studies are needed to illustrate this finding in fracture patients.

### D-dimer as a risk factor

The role of the D-dimer assay in the initial evaluation of adult patients with suspected DVT or PE has been studied extensively. In adults with a low pretest probability of DVT or PE, normal D-dimer levels indicate no major active fibrin formation and can therefore exclude these conditions, thus avoiding excessive testing (24). Clinically, we usually use it in combination with ultrasound for an exclusion diagnosis. Our study also showed that the AUC of the D-dimer level is the largest area under the curve compared to other risk factors, indicating that it has the strongest predictive power. The two highest categories of D-dimer levels (≥ 960, < 2,102; ≥ 2,102 ng/ml) increased the odds ratio of DVT by 4.215 times and 7.896 times, respectively, compared to the lowest reference category (< 490 ng/ml). This observation may be useful in the future.

### Red blood cell as risk a factor

The traditional view of blood coagulation involves only vasoconstriction, clotting factors, platelets, etc. However, abnormalities in RBC number and/or function have also been associated with DVT risk (25). RBC contributions to DVT are thought to stem from their effects on blood viscosity and margination of platelets to the vessel wall. More recent studies suggest that RBCs also express phosphatidylserine, supporting thrombin generation and decreasing fibrinolysis. RBCs contribute to blood viscosity, which increases non-linearly with hematocrit and thus represents a pathogenic mechanism for thrombosis (26). The efficacy of this diffusive exchange is determined by maximizing the active contact area between an RBC and the vessel wall. In addition to the interaction of RBCs with activated endothelial cells, they can be exposed and bind to the subendothelial matrix when the endothelium is damaged (27).

Red blood cells have a remarkably soft cytoskeleton under the plasma membrane that has a special dynamic molecular structure composed of a non-covalent association of proteins. Structural alterations of the transmembrane or cytoskeletal proteins, or of the composition of membrane phospholipids, result in rupture of the RBC membrane (haemolysis) or an increase in membrane stiffness (28). An increase in hemoglobin concentration or a decrease in hemoglobin solubility can cause changes in the viscosity of the cytoplasm. This study revealed that the number of red blood cells was correlated with DVT incidence, which supports the above research results. There were no significant differences in the hemoglobin level, mean erythrocyte hemoglobin content, mean erythrocyte hemoglobin concentration, or mean erythrocyte volume between the DVT and control groups.

### Glucose and thrombosis

Previous study by our research group illustrated the major adverse cerebral and cardiovascular events (MACCEs) of 771 patients with diabetes undergoing coronary artery bypass grafting (CABG), categorized according to BMI. The MACCEs include all-cause mortality, myocardial infarction, heart failure, cerebral infarction, and revascularization. The various BMI groups were not associated with significant differences in 5-year MACCEs (29). We also reviewed the process of hyperglycemia leading to diabetes, including the process leading from insulin resistance to diabetes, and analyzed the molecular mechanism of adipose tissue in diabetes. We also reviewed the molecular pathways initiated in situations of metabolic stress in hopes of gaining a deeper knowledge of the pathophysiology of diabetes (7).

Hyperglycemia is normally associated with a state of chronic, low-grade inflammation, which is a risk factor for thrombosis (30). Type 2 diabetes is considered a prothrombotic condition in some studies, with the hypothesized mechanism of suppressing fibrinolysis through increasing fibrinolytic inhibitor PAI-1 levels. This study first illustrated that a high preoperative glucose level was independently correlated with preoperative DVT, in addition to the usual risk factors. A previous study found endothelial dysfunction and platelet hyperactivity in the pathogenesis of atherosclerotic vascular complications, which usually refers to arterial thrombosis in type 2 diabetes mellitus (31). Another study illustrated that diabetes mellitus carried an increased risk for VTE, which is apparent only in younger patients in whom complications also increase the risk of VTE (32). However, another study found that diabetes mellitus and diabetes complications were not independent risk factors for incident VTE (33).

In the present study, we did not find an association between type 2 diabetes mellitus and DVT. No association was found between fasting glucose levels as a continuous variable and the risk of DVT. We found that preoperative fasting glucose levels, rather than type 2 diabetes mellitus, was associated with DVT in limb fracture patients. However, data on HbA1c levels were lacking. More studies on these topics should be performed.

We also found a weakly increased risk of DVT in the middle category of fasting glucose levels compared to the lowest reference category, which is different from the results of a previous study (34).

Several limitations should also be considered. First, HbA1c levels, which are a more accurate measure than fasting glucose to reflect hyperglycemia and diabetes status, were not available in this study. Second, we did not classify the lower limb fractures by fracture site due to the limited sample size, which may affect the results. Third, the number of patients in the DVT group is low. And the number of diabetic individuals was limited in both the DVT patients and controls, which limited the power of some of the subgroup analyses, especially the hyperglycemia groups.

In conclusion, we found that hyperglycemia increased the incidence of DVT in trauma patients with lower limb fracture, revealing a correlation between blood glucose and venous thrombosis. This suggests that patients with hyperglycemia should be aware of the possibility of deep vein thrombosis. More attention should be paid to this, especially for those patients in the early stages of diabetes or with impaired blood glucose regulation. More physical activity may be needed for these patients. As for the specific mechanism underpinning this correlation, further research is needed.

## Data availability statement

The data analyzed in this study is subject to the following licenses/restrictions: data protection. Requests to access these datasets should be directed to HC, chiefchu@163.com.

## Ethics statement

The studies involving human participants were reviewed and approved by the Ethics Committee of Affiliated Hospital of Qingdao University. Written informed consent was not required for this study, in accordance with the local legislation and institutional requirements.

## Author contributions

XL finished the study design and wrote the manuscript. TL and XM finished the statistics. HH, CW, and YH provided some suggestions on medication and were involved in the patient’s diagnosis management. HC supervised the study and approved the manuscript. All authors contributed to the article and approved the submitted version.
